# The Unified Form of Code Biases and Positioning Performance Analysis in Global Positioning System (GPS)/BeiDou Navigation Satellite System (BDS) Precise Point Positioning Using Real Triple-Frequency Data

**DOI:** 10.3390/s19112469

**Published:** 2019-05-30

**Authors:** Peng Liu, Honglei Qin, Li Cong

**Affiliations:** School of Electronic and Information Engineering, Beihang University, No. 37 Xueyuan Road, Beijing 100191, China; blairliu@buaa.edu.cn (P.L.); qhlmmm@sina.com (H.Q.)

**Keywords:** inter-frequency clock bias, inter-system clock bias, multi-frequency, multi-system, precise point positioning

## Abstract

Multi- system and multi-frequency are two key factors that determine the performance of precise point positioning. Both multi-frequency and multi-system lead to new biases, which are not solved systematically. This paper concentrates on mathematical models of biases, influences of these biases, and positioning performance analysis of different observation models. The biases comprise the inter-frequency clock bias in multi-frequency and the inter-system clock bias in multi-system. The former is the residual differential code biases (DCBs) from receiver clock and satellite clock and usually occurs at the third frequency, the latter is the deviation of the receiver clock errors in different systems. Unified mathematical models of the biases are presented by analyzing the general formula of observation equations. The influences of these biases are validated by experiments with corresponding observation models. Subsequently, the experiments, which are based on the data at five globally distributed stations in Multi-Global Navigation Satellite System (GNSS) Experiment (MGEX) on day of year 100, 2018, assess positioning performance of different observation models with combination of frequencies (dual-frequency or triple- frequency) and systems (BeiDou Navigation Satellite System (BDS) or Global Positioning System (GPS)). The results show that the performances of triple-frequency models are almost as the same level as the dual-frequency models. They provide scientific support for the triple-frequency ambiguity-fixed solution which has a better convergence characteristic than dual-frequency ambiguity-fixed solution. Furthermore, the biases are expressed as an unified form that gives an important and valuable reference for future research on multi-frequency and multi-system precise point positioning.

## 1. Introduction

Precise point positioning (PPP) [1,2] eliminates or weakens many errors in pseudo-range and carrier-phase to positioning solutions. However, the instrumental biases in pseudorange and carrier phase are not eliminated, and convergence time of PPP is too long–about 30 min [2]. Guo [3] suggested that users can handle this with the differential code bias (DCB) in PPP to further improve errors and convergence time.

In addition, Global Positioning System (GPS) satellites began to provide triple frequency signals [4,5,6]. The Chinese BeiDou Navigation Satellite System (BDS) is the world’s first Global Navigation Satellite System (GNSS) to provide three-frequency signal services. BDS had already launched a regional navigation service at the end of 2012 and continued to develop a global positioning service for 2018 [7,8]. Triple-frequency, which can be used to decrease the convergence time of PPP, became a research focus. Elsobeiey [9] estimated P1-C5 satellites differential code biases based on different criteria and assessed the performance of triple-PPP by processing the modernized L5 signal along with the legacy GPS signals.

With the introduction of a third frequency signal, inter-frequency clock biases (IFBs) from satellite clocks and receiver clocks among multi-frequency signals have been found. Guo et al. [10] summarized a new receiver bias named receiver IFB from P3. For the carrier phase observations, the bias can be absorbed into the ambiguity making it as a float form, resulting in a much longer convergence time and degrading the positional accuracy of PPP. Li [11] investigated a method of estimating the satellite IFB by dividing the satellite IFB into a constant and a variable part. Subsequently, the method was widely used to analyze the characteristics of the satellite IFB [12,13] and its influence for positioning [14,15,16,17]. Furthermore, Guo and Geng [18] proposed an alternative approach where a second satellite clock parameter dedicated to the L5 signals is estimated along with the legacy L1/L2 clock in the undifferenced uncombined GNSS model. It still lacks a uniform formula of IFB from both the receiver and satellites. In addition, the model with IFB derived from the receiver can not be used in the future triple-frequency ambiguity-fixed PPP.

To overcome the problem of the long convergence time of PPP, multi-system combination (which adds observations) is also an available method. Both receiver clocks and satellite clocks in any two systems are unequal, they lead to inter-system clock bias (ISB). El-Mowafy [19] reviewed various types of biases in GNSS data, including satellite and receiver hardware biases, differential code biases, differential phase biases, initial fractional phase biases, inter-system receiver time biases, and system time scale offset. Aggrey and Bisnath [20] analyzed the improvements of the float ambiguities with the biases correction by observing PPP convergence characteristics with or without bias correction in dual-frequency and triple-frequency scenarios during the first few minutes. The models lack conjoint analysis of ISB and IFB.

To solve the above bias problems, we revised the formula of the receiver IFB on a third frequency carrier phase observation to increase the fixed success rate of the third frequency ambiguity. Such a revised IFB decreases convergence time and increases the positional accuracy of PPP, compared with the model of Guo et al. [10]. Furthermore, we merged the formulae of IFB from both receiver and satellite and shed light on the mathematical meaning of ISB. Finally, we presented the observation equations of uncombined observation model with the IFB and ISB and analyzed the daily positioning performance in GPS/BDS PPP with the use of real triple-frequency data.

The remainder of this paper is organized as follows. The unified biases forms of code biases (IFB and ISB), which are the unified definitions and mathematical formulae of code biases from receiver and satellites, are respectively proposed by analyzing the original triple-frequency observation equations in Section 2. Section 3 presents and analyzes the observation equations in different models, specially uncombined observation model with triple-frequency multi-GNSS. Experiments are conducted to present the validation of the biases and the assessment of different PPP models by using the actual dual-frequency or triple-frequency data in Section 4. Finally, some conclusions are given in Section 5.

## 2. The Unified Forms of Code Biases

In this section, we present the definitions and mathematical representations of the IFB and ISB by analyzing the original uncombined observation model.

### 2.1. Basic Observation Equations

According to the uncombined observation model in Refs. [21,22,23], the original uncombined observation model with single receiver, single satellite, and single epoch is expressed by using a short general form of pseudorange P and carrier phase L as follows.

(1) $$P_{i}^{sys}=\rho^{sys}+c\left(dt-dT+bias_{P_{i}^{sys}}^{r}-bias_{P_{i}^{sys}}^{s}\right)+T_{w}+I_{i}^{sys}+\varepsilon_{P_{i}^{sys}}$$

(2) $$L_{i}^{sys}=\rho^{sys}+c\left(dt-dT+bias_{L_{i}^{sys}}^{r}-bias_{L_{i}^{sys}}^{s}\right)+T_{w}-I_{i}^{sys}-\lambda_{i}^{sys}N_{i}^{sys}+\varepsilon_{L_{1}^{sys}}$$

where *c* is speed of light, sys represents constellation identification, for instance, GPS or BDS. $P_{i}^{sys}$ is the pseudorange in frequency i at constellation sys, $L_{i}^{sys}$ is the carrier phase in frequency i at constellation sys. $\rho^{sys}$ is the distance between station and satellite at constellation sys, dt is receiver clock error, dT is satellite clock error, Tw is the projection of tropospheric zenith wet path delay, $I_{i}^{sys}$ is ionospheric delay in $P_{i}$ at constellation sys. The system frequency ratio $\gamma_{i}^{sys}=f_{1,sys}^{2}/f_{i,sys}^{2}$, where $f_{i,sys}$ is the frequency of signal *i* at constellation sys. $N_{i}^{sys}$ is the ambiguity of carrier phase $L_{i}^{sys}$. $bias_{P_{IF}^{sys}}^{r}$, $bias_{P_{IF}^{sys}}^{s}$, $bias_{L_{i}^{sys}}^{r}$, and $bias_{L_{i}^{sys}}^{s}$ are hardware delay in pseudorange $P_{i}^{sys}$ and carrier phase $L_{i}^{sys}$. $\varepsilon_{P_{i}^{sys}}$ and $\varepsilon_{L_{i}^{sys}}$ are observation noise in pseudorange $P_{i}^{sys}$ and carrier phase $L_{i}^{sys}$, respectively.

Because satellite clock errors degrade the accuracy of PPP and can not be estimated directly, the clock product provided by International GNSS Service (IGS) organization is used to correct satellite clock errors. For legacy GPS, it has been a common practice to define clock offsets in precise ephemeris products with respect to an ionosphere-free dual-frequency combination of conventional reference signals ($P_{1}^{GPS}$ and $P_{2}^{GPS}$) [24]. According to the BDS Interface Control Document (ICD), currently BDS adopts the precise ephemeris (and clock) products referring to the $P_{1}^{BDS}/P_{2}^{BDS}$ ionosphere-free combination of dual-frequency observations [25,26]. When the satellite clock products computed by using other combined signals or single signal differing from the conventional reference signal $P_{1}^{sys}/P_{2}^{sys}$ are employed in GNSS applications, the pseudoranges are well known to be affected by instrumental biases [27,28]. Therefore, these satellite clock biases, which are inconsistent with satellite clock products, should be corrected by differential code biases (DCBs) for pseudorange-based positioning, timing or ionosphere modeling [29]. The differential code biases (DCBs) provided by GNSS communities particularly for the post-processing PPP applications are as the same physical significance as timing group delays (TGDs) and inter-signal corrections (ISCs) in broadcast ephemeris, but DCBs are more accurate than TGDs and ISCs [30,31].

The hardware delays can not be estimated, because they can not be separated from the receiver clock and ambiguity respectively. The relationship between hardware delays and DCBs is as follows.

(3) $$\begin{array}{cc} DCB_{P_{i}{P_{j}}^{sys}}^{r} & =bias_{P_{i}^{sys}}^{r}-bias_{P_{j}^{sys}}^{r},\;i\neq j,\;and\;i,j=1,2,3 \end{array}$$

(4) $$\begin{array}{cc} DCB_{P_{i}{P_{j}}^{sys}}^{s} & =bias_{P_{i}^{sys}}^{s}-bias_{P_{j}^{sys}}^{s},\;i\neq j,\;and\;i,j=1,2,3 \end{array}$$

where $DCB_{P_{i}{P_{j}}^{sys}}^{r}$ and $DCB_{P_{i}{P_{j}}^{sys}}^{s}$ are difference code biases between $P_{i}$ and $P_{j}$ from receiver and satellite.

When clock errors from receiver and satellite are based on conventional ionosphere-free model with dual-frequency (CIF2), they can be indicated as follows.

(5) $$\begin{array}{cc} dt_{P_{CIF2}^{sys}} & =dt_{P_{}^{sys}}+\alpha bias_{P_{1}^{sys}}^{r}+\beta bias_{P_{2}^{sys}}^{r} \end{array}$$

(6) $$\begin{array}{cc} dT_{P_{CIF2}^{sys}} & =dT_{P_{}^{sys}}+\alpha bias_{P_{1}^{sys}}^{s}+\beta bias_{P_{2}^{sys}}^{s} \end{array}$$

According to Formulas (1)–(6), the dual-frequency observation equations are converted as follows.

(7) $$\begin{array}{cc} P_{1}^{sys}= & \rho^{sys}+cdt_{P_{CIF2}^{sys}}-cdT_{P_{CIF2}^{sys}}+T_{w}+I_{1}^{sys}+c\beta^{sys}DCB_{P1P2^{sys}}^{r}-c\beta^{sys}DCB_{P1P2^{sys}}^{s}+\varepsilon_{P_{1}^{sys}} \end{array}$$

(8) $$\begin{array}{cc} P_{2}^{sys}= & \rho^{sys}+cdt_{P_{CIF2}^{sys}}-cdT_{P_{CIF2}^{sys}}+T_{w}+\gamma_{2}^{sys}\left(I_{1}^{sys}+c\beta^{sys}DCB_{P1P2^{sys}}^{r}-c\beta^{sys}DCB_{P1P2^{sys}}^{s}\right)+\varepsilon_{P_{2}^{sys}} \end{array}$$

(9) $$\begin{array}{cc} L_{1}^{sys}= & \rho^{sys}+cdt_{P_{CIF2}^{sys}}-cdT_{P_{CIF2}^{sys}}+T_{w}-\left(I_{1}^{sys}+c\beta^{sys}DCB_{P1P2^{sys}}^{r}-c\beta^{sys}DCB_{P1P2^{sys}}^{s}\right)\\ & -\lambda_{1}^{sys}\left(N_{1}^{sys}-f_{1,sys}bias_{1}^{sys}\right)+\varepsilon_{L_{1}^{sys}} \end{array}$$

(10) $$\begin{array}{cc} L_{2}^{sys}= & \rho^{sys}+cdt_{P_{CIF2}^{sys}}-cdT_{P_{CIF2}^{sys}}+T_{w}-\gamma_{2}^{sys}\left(I_{1}^{sys}+c\beta^{sys}DCB_{P1P2^{sys}}^{r}-c\beta^{sys}DCB_{P1P2^{sys}}^{s}\right)\\ & -\lambda_{2}^{sys}\left(N_{2}^{sys}-f_{2,sys}bias_{2}^{sys}\right)+\varepsilon_{L_{2}^{sys}} \end{array}$$

where $\alpha^{sys}=f_{1,sys}^{2}/\left(f_{1,sys}^{2}-f_{2,sys}^{2}\right)$, $\beta^{sys}=-f_{2,sys}^{2}/\left(f_{1,sys}^{2}-f_{2,sys}^{2}\right)$, and $bias_{i}^{sys}=2\beta^{sys}\gamma_{i}^{sys}\left(DCB_{P1P2^{sys}}^{r}-DCB_{P1P2^{sys}}^{s}\right)+bias_{L_{i}^{sys}}^{r}-bias_{L_{i}^{sys}}^{s}-bias_{P_{i}^{sys}}^{r}+bias_{P_{i}^{sys}}^{s}$.

### 2.2. Inter Frequency Clock Bias

If we compare with double-frequency, the triple-frequency model add a new carrier signal to the observations. The triple-frequency observation equations are illustrated as follows.

(11) $$\begin{array}{cc} P_{1}^{sys}= & \Delta \rho^{sys}+\delta I^{sys}+\varepsilon_{P_{1}^{sys}} \end{array}$$

(12) $$\begin{array}{cc} P_{2}^{sys}= & \Delta \rho^{sys}+\gamma_{2}^{sys}\delta I^{sys}+\varepsilon_{P_{2}^{sys}} \end{array}$$

(13) $$\begin{array}{cc} P_{3}^{sys}= & \Delta \rho^{sys}+\gamma_{3}^{sys}\delta I^{sys}-c\left(\beta^{sys}\left(\gamma_{3}^{sys}-1\right)DCB_{P1P2^{sys}}^{r}+DCB_{P1P3^{sys}}^{r}\right)\\ & +c\left(\beta^{sys}\left(\gamma_{3}^{sys}-1\right)DCB_{P1P2^{sys}}^{s}+DCB_{P1P3^{sys}}^{s}\right)+\varepsilon_{P_{3}^{sys}} \end{array}$$

(14) $$\begin{array}{cc} L_{1}^{sys}= & \Delta \rho^{sys}-\delta I^{sys}-\lambda_{1}^{sys}\delta N_{1}^{sys}+\varepsilon_{L_{1}^{sys}} \end{array}$$

(15) $$\begin{array}{cc} L_{2}^{sys}= & \Delta \rho^{sys}-\gamma_{2}^{sys}\delta I^{sys}-\lambda_{2}^{sys}\delta N_{2}^{sys}+\varepsilon_{L_{2}^{sys}} \end{array}$$

(16) $$\begin{array}{cc} L_{3}^{sys}= & \Delta \rho^{sys}-\gamma_{3}^{sys}\delta I^{sys}-c\left(\beta^{sys}\left(\gamma_{3}^{sys}-1\right)DCB_{P1P2^{sys}}^{r}+DCB_{P1P3^{sys}}^{r}\right)\\ & +c\left(\beta^{sys}\left(\gamma_{3}^{sys}-1\right)DCB_{P1P2^{sys}}^{s}+DCB_{P1P3^{sys}}^{s}\right)-\lambda_{3}^{sys}\delta N_{3}^{sys}+\varepsilon_{L_{3}^{sys}} \end{array}$$

where $\Delta \rho^{sys}=\rho^{sys}+cdt_{P_{CIF2}^{sys}}-cdT_{P_{CIF2}^{sys}}+T_{w}$, $\delta I^{sys}=I_{1}^{sys}+c\beta^{sys}DCB_{P1P2^{sys}}^{r}-c\beta^{sys}DCB_{P1P2^{sys}}^{s}$, and $\delta N_{i}^{sys}=N_{i}^{sys}-f_{i,sys}bias_{i}^{sys}$.

The DCBs in the first and second frequency are eliminated through merging transformation. When the same operation happens in the third frequency, there are residual DCBs in third frequency pseudorange $P_{3}$ and carrier phase $L_{3}$. All residual DCBs in third frequency pseudorange $P_{3}$ can be estimated as a parameter called inter frequency clock bias (IFB), which can be expressed as a function of the DCBs ($DCB_{P_{1}-P_{2}}$ and $DCB_{P_{1}-P_{3}}$). To keep physical significances of both the estimated ionospheric delay and fractional cycle bias (FCB) in the ambiguities consistent in different frequencies, IFB is also introduced into third frequency carrier phase $L_{3}$.

(17) $$IFB_{sys}=c\left(\beta^{sys}\left(\gamma_{3}^{sys}-1\right)DCB_{P1P2^{sys}}^{s}+DCB_{P1P3^{sys}}^{s}\right)-c\left(\beta^{sys}\left(\gamma_{3}^{sys}-1\right)DCB_{P1P2^{sys}}^{r}+DCB_{P1P3^{sys}}^{r}\right)$$

According to the type of the terminals (receiver, satellite), IFB can be divided into satellite IFB $IFB_{sys}^{s}$ and receiver IFB $IFB_{sys}^{r}$ as follows.

(18) $$IFB_{sys}^{s}=c\left(\beta^{sys}\left(\gamma_{3}^{sys}-1\right)DCB_{P1P2^{sys}}^{s}+DCB_{P1P3^{sys}}^{s}\right)$$

(19) $$IFB_{sys}^{r}=c\left(\beta^{sys}\left(\gamma_{3}^{sys}-1\right)DCB_{P1P2^{sys}}^{r}+DCB_{P1P3^{sys}}^{r}\right)$$

When IFB is regarded as variable, it can be estimated according to the references [11,14,15,16,17]. The root mean square (RMS) values of IFBs in GPS and BDS are less than 0.3 mm [18,32]. They are far less than the RMS value of noise in carrier phase (3 mm) [10]. We ignore time-variant component of IFB and regard IFB as a constant [19]. The satellite IFB needs to be corrected using the DCB files published by IGS organization, and the receiver IFB needs to be estimated together with other unknown parameters. Namely, the estimated IFB in the actual PPP is the receiver component of IFB.

(20) $$IFB_{sys}^{estimated}=-IFB_{sys}^{r}=-c\left(\beta^{sys}\left(\gamma_{3}^{sys}-1\right)DCB_{P1P2^{sys}}^{r}+DCB_{P1P3^{sys}}^{r}\right)$$

### 2.3. Inter System Clock Bias

Similarly, in multi-systems, due to the hardware delays ($bias_{P_{i}^{sys}}^{r}$ and $bias_{P_{i}^{sys}}^{s}$) between the two systems being unequal, the reference receiver clock errors are unequal between the two systems, and the satellite clock has been corrected by using the DCB files published by the IGS organization. Compared with the reference system (GPS in the experiment), the observation equations in another system (BDS in the experiment) are as follows.

(21) $$\begin{array}{cc} P_{1}^{sys}= & \rho^{sys}+cdt_{P_{CIF2}^{ref}}-cdT_{P_{CIF2}^{sys}}+T_{w}+\delta I^{sys}\\ & +c\left(\alpha^{sys}bias_{P_{1}^{sys}}^{r}+\beta^{sys}bias_{P_{2}^{sys}}^{r}-\left(\alpha^{ref}bias_{P_{1}^{ref}}^{r}+\beta^{ref}bias_{P_{2}^{ref}}^{r}\right)\right)+\varepsilon_{P_{1}^{sys}} \end{array}$$

(22) $$\begin{array}{cc} P_{2}^{sys}= & \rho^{sys}+cdt_{P_{CIF2}^{ref}}-cdT_{P_{CIF2}^{sys}}+T_{w}+\gamma_{2}^{sys}\delta I^{sys}\\ & +c\left(\alpha^{sys}bias_{P_{1}^{sys}}^{r}+\beta^{sys}bias_{P_{2}^{sys}}^{r}-\left(\alpha^{ref}bias_{P_{1}^{ref}}^{r}+\beta^{ref}bias_{P_{2}^{ref}}^{r}\right)\right)+\varepsilon_{P_{2}^{sys}} \end{array}$$

(23) $$\begin{array}{cc} L_{1}^{sys}= & \rho^{sys}+cdt_{P_{CIF2}^{ref}}-cdT_{P_{CIF2}^{sys}}+T_{w}-\delta I^{sys}-\lambda_{1}^{sys}\delta N_{1}^{sys}\\ & +c\left(\alpha^{sys}bias_{P_{1}^{sys}}^{r}+\beta^{sys}bias_{P_{2}^{sys}}^{r}-\left(\alpha^{ref}bias_{P_{1}^{ref}}^{r}+\beta^{ref}bias_{P_{2}^{ref}}^{r}\right)\right)+\varepsilon_{L_{1}^{sys}} \end{array}$$

(24) $$\begin{array}{cc} L_{2}^{sys}= & \rho^{sys}+cdt_{P_{CIF2}^{ref}}-cdT_{P_{CIF2}^{sys}}+T_{w}-\gamma_{2}^{sys}\delta I^{sys}-\lambda_{2}^{sys}\delta N_{2}^{sys}\\ & +c\left(\alpha^{sys}bias_{P_{1}^{sys}}^{r}+\beta^{sys}bias_{P_{2}^{sys}}^{r}-\left(\alpha^{ref}bias_{P_{1}^{ref}}^{r}+\beta^{ref}bias_{P_{2}^{ref}}^{r}\right)\right)+\varepsilon_{L_{2}^{sys}} \end{array}$$

where $\delta \rho^{sys}=\rho^{sys}+cdt_{P_{CIF2}^{ref}}-cdT_{P_{CIF2}^{sys}}+T_{w}$. Compare with reference system, there is a receiver clock error compensation in another system called inter system clock bias (ISB) as follows. The estimated ISB in the actual PPP is the receiver component of ISB.

(25) $$ISB_{sys-ref}=cdt_{P_{CIF2}^{sys}}-cdt_{P_{CIF2}^{ref}}=c\left(\alpha^{sys}bias_{P_{1}^{sys}}^{r}+\beta^{sys}bias_{P_{2}^{sys}}^{r}-\alpha^{ref}bias_{P_{1}^{ref}}^{r}-\beta^{ref}bias_{P_{2}^{ref}}^{r}\right)$$

## 3. Observation Models in Single or Multi-GNSS

### 3.1. Uncombined Observation Model with Triple-Frequency Multi-GNSS

There are IFB and ISB in triple-frequency multi-system, where the observation equations can be illustrated by the following formula.

(26) $$P_{1}^{sys}=\delta \rho^{sys}+\delta I^{sys}+ISB_{sys-ref}+\varepsilon_{P_{1}^{sys}}$$

(27) $$P_{2}^{sys}=\delta \rho^{sys}+\gamma_{2}^{sys}\delta I^{sys}+ISB_{sys-ref}+\varepsilon_{P_{2}^{sys}}$$

(28) $$P_{3}^{sys}=\delta \rho^{sys}+\gamma_{3}^{sys}\delta I^{sys}+IFB_{sys}+ISB_{sys-ref}+\varepsilon_{P_{3}^{sys}}$$

(29) $$L_{1}^{sys}=\delta \rho^{sys}-\delta I^{sys}-\lambda_{1}^{sys}\delta N_{1}^{sys}+ISB_{sys-ref}+\varepsilon_{L_{1}^{sys}}$$

(30) $$L_{2}^{sys}=\delta \rho^{sys}-\gamma_{2}^{sys}\delta I^{sys}-\lambda_{2}^{sys}\delta N_{2}^{sys}+ISB_{sys-ref}+\varepsilon_{L_{2}^{sys}}$$

(31) $$L_{3}^{sys}=\delta \rho^{sys}-\gamma_{3}^{sys}\delta I^{sys}+IFB_{sys}+ISB_{sys-ref}-\lambda_{3}^{sys}\delta N_{3}^{sys}+\varepsilon_{L_{3}^{sys}}$$

According to the above formulae, the hardware delays ($bias_{P_{i}^{sys}}^{r}$, $bias_{P_{i}^{sys}}^{s}$, $bias_{L_{i}^{sys}}^{r}$, and $bias_{L_{i}^{sys}}^{s}$) are absorbed by ionospheric delay $I_{1}^{sys}$ and ambiguity $N_{i}^{sys}$. The estimated ionospheric delay $\delta I^{sys}$ combines ionospheric delay and some hardware delays. The estimated ionospheric delay can be also eliminated by ionosphere-free models. The estimated ambiguities $\delta N_{i}^{sys}$ are the combination of float ambiguities and some hardware delays. These hardware delays can influence the integer feature of ambiguities. In ambiguity-fixed PPP, FCB or uncalibrated phase delay (UPD), provided by the Continuously Operating Reference Stations (CORS) network, corrects the non-integer estimated ambiguities $\delta N_{i}^{sys}$ to integer estimated ambiguities. It means the observation equation in the third frequency carrier phase $L_{3}$ must contain IFB, namely, it must follow Formula (16).

### 3.2. Other Typical Observation Models

In PPP, the most widely used models are conventional ionosphere-free model (CIF) and uncombined model (UC), including uncombined model with triple-frequency (UC3), uncombined model with dual-frequency (UC2), conventional ionosphere-free model with triple-frequency (CIF3), conventional ionosphere-free model with dual-frequency (CIF2). The Table 1 summaries the differences of the models, where each equation in CIF has IFB or no IFB. Namely, IFBs in CIF can not be divided from receiver clock, there is no IFB in estimated parameters.

The observation equations in CIF are respectively either the combining pseudoranges or the combining carrier phases as follows.

(32) $$P_{CIF3}^{sys}=k_{1,P}P_{1}^{sys}+k_{2,P}P_{2}^{sys}+k_{3,P}P_{3}^{sys}$$

(33) $$L_{CIF3}^{sys}=k_{1,L}L_{1}^{sys}+k_{2,L}L_{2}^{sys}+k_{3,L}L_{3}^{sys}$$

where $k_{1,P}$, $k_{2,P}$, $k_{3,P}$, $k_{1,L}$, $k_{2,L}$, and $k_{3,L}$ are combinging coefficients of the pseudorange and carrier phase. The six coefficients satisfy the following Formula (34) to make sure the pseudorange and carrier phase measurements have the properties of geometry preserving, ionosphere-free, and the lowest noise propagation. The coefficients of the pseudorange are equal to the coefficients of the carrier phase. Table 2 gives dual-frequency the specific values of coefficients for GPS and BDS systems.

(34) $$\left\{\begin{array}{c} k_{1}+k_{2}+k_{3}=1\\ k_{1}+k_{2}f_{1}^{2}/f_{2}^{2}+k_{3}f_{1}^{2}/f_{3}^{2}=0\\ k_{1}^{2}+k_{2}^{2}+k_{3}^{2}=MIN \end{array}\right.$$

## 4. Analysis And Assessment

### 4.1. Experimental Strategy

#### 4.1.1. Configuration Strategy

For the sake of improving the accuracy and convergence time, some errors in PPP can be corrected by corresponding methods. All error corrections are shown in Table 3.

Our self-developed MATLAB processing software based on Prototyping RTKLIB2.4.3 is used to perform multi-GNSS globally distributed reference network experiments. The initial values and corresponding variance-covariance of XYZ and dt are for the least squares solution in the first epoch. The initial value of Tw is calculated by the Hopfield model in first epoch, the corresponding variance-covariance is set to arbitrary value (0.25 in experiment). $N_{i}$ and *I* are derived from undifferenced observation equations [35].

The noises of pseudorange and carrier phase are usually set to 0.3 m and 0.003 m [10], the weight ratio of pseudorange and carrier phase is 100. And the variance of dynamic noise in receiver clock error is 900 m${}^{2}$/s${}^{2}$, the variance of dynamic noise in troposphere Zenith wet path delay is 10${}^{-8}$ m${}^{2}/$s${}^{2}$, the variance of dynamic noise in ionosphere is 10${}^{-6}$m${}^{2}$/s${}^{2}$ [36].

The verification used daily (24 h) solutions in day of year (DOY) 100, 2018. General settings adopted for the PPP validation are provided in Table 4. The igs14atx file provided by International GNSS Service (IGS) only have the Antenna Phase Center Variations (PCVs) and Antenna Phase Center Offsets (PCOs) on first two frequencies (L1/L2) in GPS. The PCV and PCO on the third frequency L5 in GPS can not be gotten directly. The L5 frequency (1176.45 Mhz) is approximate to the L2 frequency (1227.6 Mhz), we simple presume that the L5 frequency shares the same PCOs/PCVs as those on the L2 frequency [18]. Meanwhile, the PCV and PCO in BDS are set to constant zero and [0.6, 0, 1.1], respectively [37].

**Figure 1:**
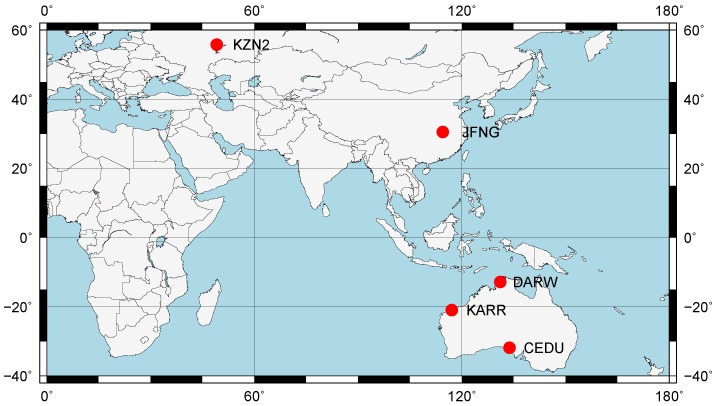
Distribution of the stations used in this experiment.

There are 146 globally distributed reference stations at MGEX, of which 39 stations have the coordinates in the file gbm19962.clk. From the 39 globally distributed reference stations, we chose five stations (CEDU, DARW, JFNG, KARR, KZN2) which are shown in Figure 1. These stations are mostly concentrated in the Asia-Pacific region where the number of satellites with triple-frequency data is sufficient.

#### 4.1.2. Availability of Multi-Frequency Models

**Figure 2:**
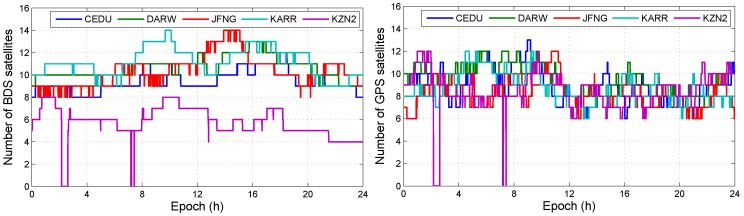
The number of BDS/GPS satellite with dual-frequency observation data.

**Figure 3:**
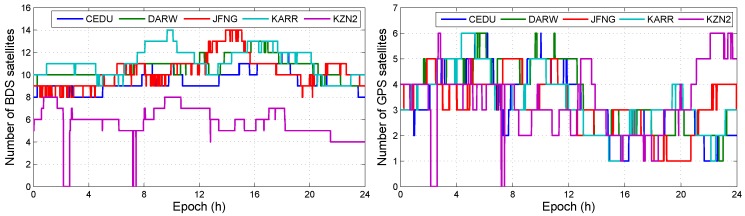
The number of BDS/GPS satellite with triple-frequency observation data.

Not all BDS/GPS satellites have third frequency signals. The number of BDS/GPS satellites with dual-frequency data is not equal to the number of BDS/GPS satellites with triple-frequency data. We illustrate the number of BDS or GPS satellites with dual-frequency or triple-frequency in DOY 100, 2018, which are shown in Figure 2 and Figure 3, respectively.

The results show that the number of GPS satellites with triple-frequency data is less than five on most of the time. Except the triple-frequency GPS-only, the triple-frequency BDS/GPS, the triple-frequency BDS-only, the dual-frequency BDS/GPS, the dual-frequency BDS-only, and the dual-frequency GPS-only can be used to realize daily precise point positioning. The triple-frequency data causes IFB bias and the BDS/GPS combination causes ISB bias. Before we process data by using different observation models, we analyzed the influence of ISB and IFB on positioning errors.

### 4.2. Influence of Code Biases

#### 4.2.1. Influence of IFB Bias

**Figure 4:**
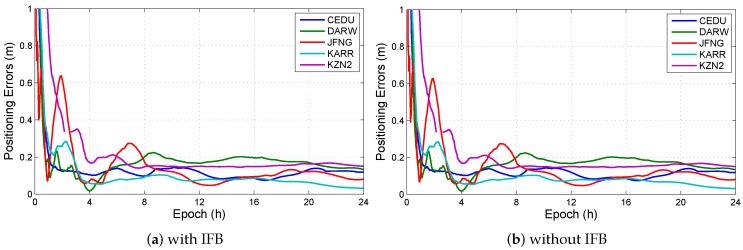
Positioning errors with or without IFB in UC3-BDS at different stations. (**a**) The positioning errors in UC3-BDS with IFB; (**b**) The positioning errors in UC3-BDS without IFB.

**Figure 5:**
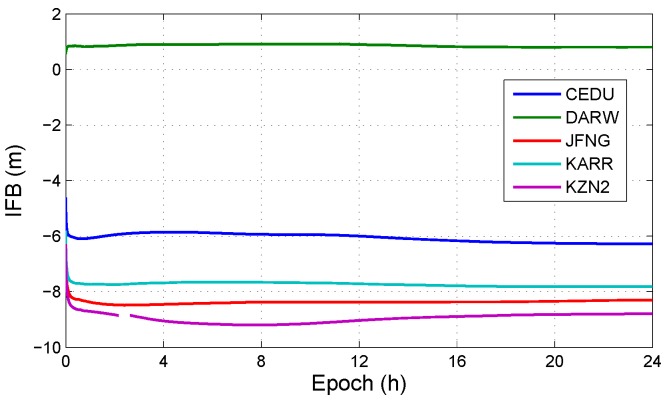
IFB at different stations in DOY 100, 2018.

In all observation models with BDS/GPS, the model, which has IFB and do not have ISB, is the uncombined model with triple-frequency BDS-only (UC3-BDS). The positioning errors in UC3-BDS are shown in Figure 4, and Figure 5 illustrates the IFB in UC3-BDS. The results show that differences in positioning errors are small. The reason is that the IFB in third frequency carrier phase $L_{3}$ is absorbed by ambiguity. The IFB in third frequency pseudorange $P_{3}$ has little effect on the positioning results, because the weight of pseudorange is small. Meanwhile, the results may be effected by the precise satellite orbit and clock products in BDS. But IFB can not be eliminated from estimated parameters. If IFB is eliminated, the third frequency ambiguity absorbs the IFB, then the convergence time and fixed success rate of the third frequency ambiguity will be not good for triple-frequency ambiguity-fixed PPP. IFB can not be omitted as a parameter to be solved. As a part of receiver DCB, each constellation has a corresponding IFB. According to the Section 2.2, each IFB is considered as a constant, the initial values of IFBs are one, the initial variances of dynamic noise in IFBs are zero.

#### 4.2.2. Influence of ISB Bias

**Figure 6:**
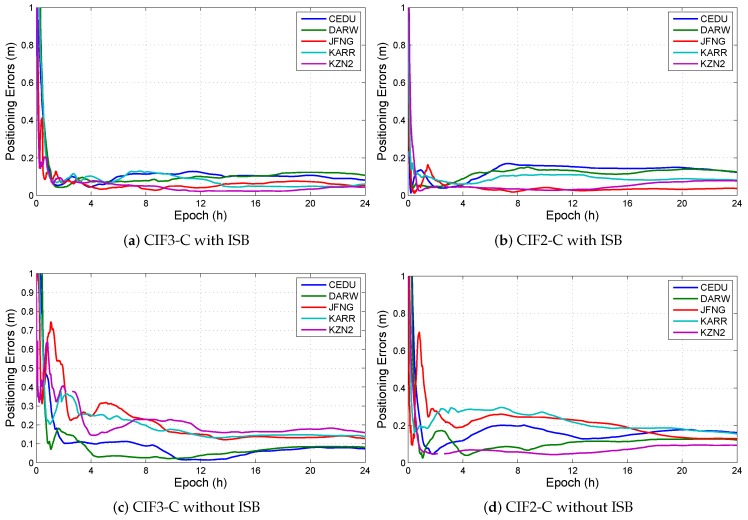

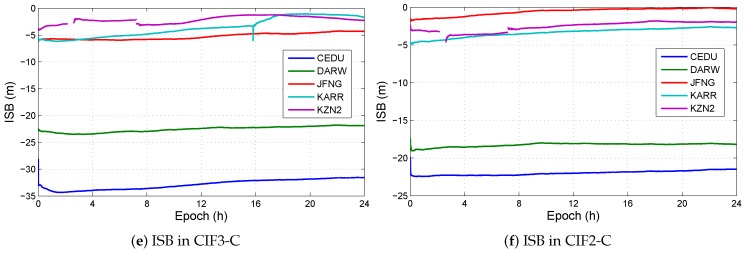
Positioning errors with or without ISB in CIF3-C and CIF2-C at different stations. (**a**) The positioning errors in CIF3-C with ISB; (**b**) The positioning errors in CIF2-C with ISB; (**c**) The positioning errors in CIF3-C without ISB; (**d**) The positioning errors in CIF2-C without ISB; (**e**) The ISB in CIF3-C, and (**f**) The ISB in CIF2-C.

In all observation models with BDS/GPS, the models, which have ISB and do not have IFB, include conventional ionosphere-free model with triple-frequency BDS/GPS combination (CIF3-C) and conventional ionosphere-free model with dual-frequency BDS/GPS combination (CIF2-C). The positioning errors in CIF3-C and CIF2-C are shown in Figure 6. The results show that ignoring ISB can increase positioning errors in the decimeter range. ISB can not be omitted as a parameter to be solved. There is a parameter ISB between any two of systems. In this experiment, ISB is the difference between receiver clock errors in BDS and GPS. ISB is set to a random process like receiver clock error, so the variance of dynamic noise in ISB is the sum of the variances of dynamic noise in receiver clock errors from GPS and BDS. We presumed that the variances of dynamic noise in receiver clock errors from GPS and BDS are same. The variance of dynamic noise in ISB is twice than the variance of dynamic noise in receiver clock error. The initial value of ISB is arbitrary value, it is set to be one in this experiment.

#### 4.2.3. Influence of Both IFB and ISB Biases

**Figure 7:**
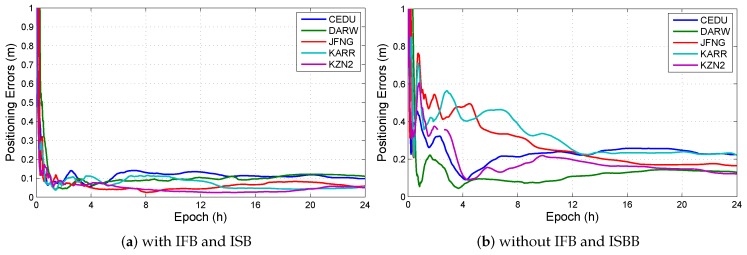
Positioning errors with or without IFB and ISB in UC3-C at different stations. (**a**) The positioning errors in UC3-C with IFB and ISB; (**b**) The positioning errors in UC3-BDS without IFB and ISB.

**Figure 8:**
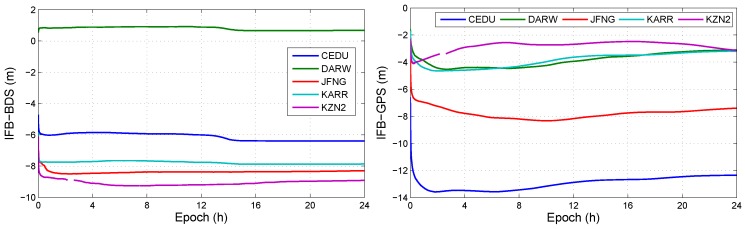
IFB at different stations in DOY 100, 2018.

**Figure 9:**
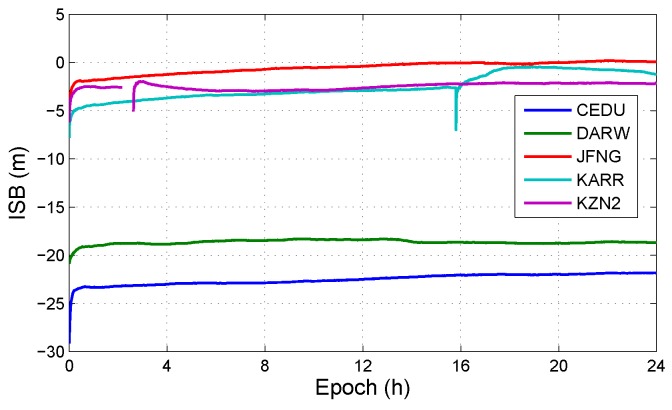
ISB at different stations in DOY 100, 2018.

This section analyzes the influence of both IFB and ISB on positioning solution. In all observation models with BDS/GPS, the models, which have IFB and do have ISB, is the uncombined model with triple-frequency BDS/GPS combination (UC3-C). The positioning errors in UC3-C are shown in Figure 7. And Figure 8 shows the $IFB_{BDS}$ and $IFB_{GPS}$ in UC3-C. Furthermore, Figure 9 shows the ISB in UC3-C.

The results show that ignoring IFB and ISB can increase positioning errors, IFB and ISB are the indispensable parameters in positioning solution.

### 4.3. Results and Discussion of Single and Multi-GNSS PPP

We will comment on the performance of positioning solutions in triple-frequency and dual-frequency models. Except for the above models (UC3-C, CIF3-C, CIF2-C, and UC3-BDS), we chose the other three models, which are the conventional ionosphere-free model with triple-frequency BDS-only (CIF3-BDS), the conventional ionosphere-free model with dual-frequency BDS-only (CIF2-BDS), and the conventional ionosphere-free model with dual-frequency GPS-only (CIF2-GPS). Furthermore, we present an extended CIF2-C model (ECIF2-C) to compare with triple-frequency models under the same satellites condition. The ECIF2-C is similar to CIF2-C, the only difference is the satellites in each epoch. The satellites, which can be used for positioning solution in CIF2-C, are determined by dual-frequency data in each epoch. Namely, for a satellite in an epoch, only if all the data in two frequencies exist, the satellite can be considered as an eligible satellite for positioning solution. The eligible satellites in ECIF2-C are determined based on triple-frequency data in each epoch. The number of the eligible satellites in ECIF2-C is less than that in CIF2-C, but the eligible satellites in ECIF2-C are the same with triple-frequency models.

**Figure 10:**
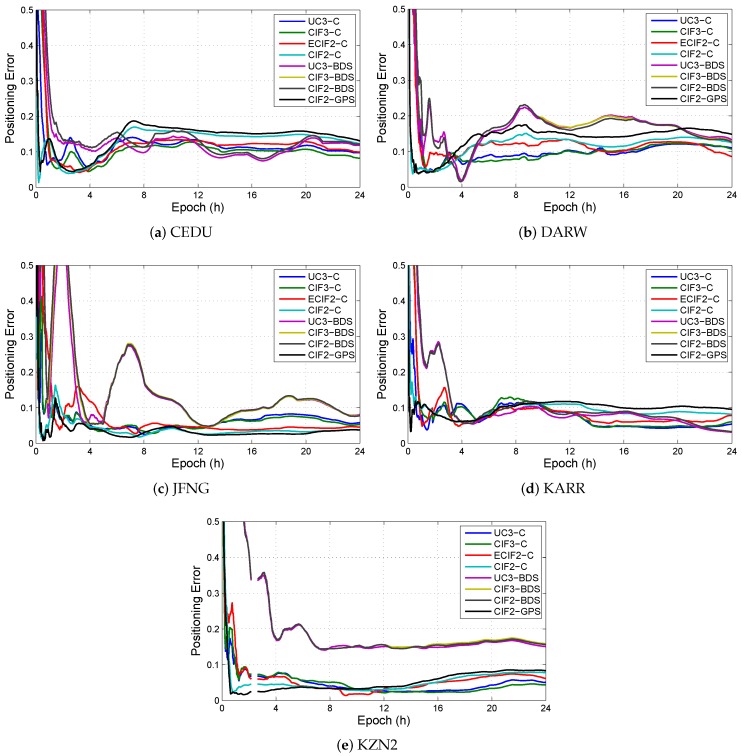
The positioning errors in different combined models for the daily solution at different stations. (**a**) The positioning errors at CEDU; (**b**) The positioning errors at DARW; (**c**) The positioning errors at JFNG; (**d**) The positioning errors at KARR; and (**e**) The positioning errors at KZN2.

As shown in Figure 10, all eight models are used to compare positioning solution at different stations (CEDU, DARW, JFNG, KARR, KZN2). The results show that the positioning errors in different models are similar, except UC3-BDS and CIF3-BDS at KZN2. Because the number of visible BDS satellites at KZN2 is less than others stations in Figure 3.

For analyzing the direction of positioning errors, Table 5 and Table 6 respectively illustrate accuracies and precisions for different combined models with the daily solution in North, East, Up directions at different stations. The results show that horizontal components in positioning errors are similar, the vertical components in positioning errors have more deviations than horizontal components, especially UC3-BDS, CIF3-BDS and CIF2-BDS. Choy et al. [38] had concluded the horizontal positioning components are more accurate than the vertical component based on much research. And the positioning errors caused by lacking satellites are concentrated on vertical components, this is very well known in GPS analysis. The horizontal positioning errors in different models are similar. When the visible satellites are sufficient, all eight models give similar estimates for positioning solution, such as UC3-C, CIF3-C, and CIF2-C.

**Figure 11:**
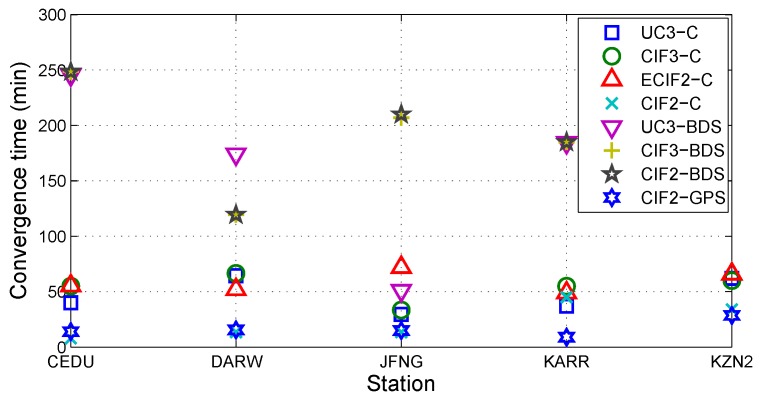
The convergence time for different combined models at different stations.

Figure 11 shows the convergence time for different combined models at different stations. If the state that the positioning errors in three directions are less than 0.1 m can hold more than 20 epochs, positioning solution is considered to converge from the beginning of this state. The convergence time is the duration from the beginning epoch of positioning process to the beginning epoch of this state. Because the vertical positioning errors are larger than the horizontal positioning errors for each epoch, the convergence time is determined by the vertical positioning errors. Because the positioning errors of vertical components are quite different in different models, the convergence time for different models are widely different. The convergence time for UC3-BDS, CIF3-BDS and CIF2-BDS is longer than others, especially at KZN2.

Table 7 shows the mean accuracy, mean precision, mean convergence time, and median convergence time for different combined models with the daily solution at different stations. The results show that CIF2-C needs more mean convergence time than CIF2-GPS. It is inconsistent with the usual result “CIF2-C usually needs less convergence time than CIF2-GPS”. In Figure 11, CIF2-C needs more mean convergence time than CIF2-GPS only occurs at KARR. Mean convergence time can not reflect convergence characteristics of different models. Median convergence time can be used as the statistic of convergence time. In Table 7, CIF2-C needs less median convergence time than CIF2-GPS in consistency with the general rule “convergence time in a multi-system PPP is less than that in a single system PPP [38]”.

## 5. Conclusions

Choosing an appropriate observation model is a critical prerequisite for positioning solutions. Adding a third frequency signal makes observation models diverse. With the introduction of a triple-frequency signal and multi-system, the IFB and ISB biases are proposed. In order to know how we can process the biases and how many benefits we can get from the new signal and multi-system, the below research was done.

According to the first-order Taylor expansion of the general observation equations, the unified definitions and mathematical formulae of code biases (IFB and ISB) from receiver and satellites were respectively derived. ISB and IFB can be better understood and calculated for the future research on triple-frequency PPP or multi-system PPP. Subsequently, the uncombined observation model with triple-frequency and multi-system was presented. Compared with the conventional model, this model takes more advantage of ambiguity-fixed PPP. Thereafter, the characteristics of basic triple-frequency observation models were theoretically analyzed to shed light on the differences of observations, parameters, redundancies, and noises.

Furthermore, the influences of ISB and IFB for positioning solution were analyzed by the PPP experiment in the CIF2-C, CIF3-C, UC3-BDS, and UC3-C at five stations. The results show that ignoring ISB can increase positioning errors in the decimeter range. While IFB does not cause many positioning errors, the ignored IFB can be absorbed by ambiguities. So ignoring IFB is not good for the fixed success rate of ambiguities. Therefore, neither IFB nor ISB could be omitted as a parameter to be solved in positioning solution. Finally, the PPP experiment with the eight observation models at five stations in DOY 100, 2018 was realized to analyze positioning performance of dual-frequency models and triple-frequency models. The results show that the triple-frequency positioning solutions are approximate to the dual-frequency positioning solutions. It provides scientific support for the ambiguity-fixed PPP using real triple-frequency data.

## Figures and Tables

**Table 1:** Precise point positioning (PPP) Models comparison with multi-satellites n and multi-systems m. Uncombined model with triple-frequency (UC3), uncombined model with dual-frequency (UC2), conventional ionosphere-free model with triple-frequency (CIF3), conventional ionosphere-free model with dual-frequency (CIF2), inter-system clock bias (ISB), inter-frequency clock bias (IFB).

Model	System	Observations	Parameters	Parameter Description
UC3	Single	6n	4n+5+1	X, Y, Z, dt, Tw, IFB, n*(*I*,$N_{1}$,$N_{2}$,$N_{3}$)
	Multi	6n	4n+5+m+1	X, Y, Z, dt, Tw, ISB, m*IFB, n*(*I*,$N_{1}$,$N_{2}$,$N_{3}$)
CIF3	Single	2n	n+5	X, Y, Z, dt, Tw, n*N
	Multi	2n	n+5+1	X, Y, Z, dt, Tw, ISB, n*N
UC2	Single	4n	3n+5	X, Y, Z, dt, Tw, n*(*I*,$N_{1}$,$N_{2}$)
	Multi	4n	3n+5+1	X, Y, Z, dt, Tw, ISB, n*(*I*,$N_{1}$,$N_{2}$)
CIF2	Single	2n	n+5	X, Y, Z, dt, Tw, n*N
	Multi	2n	n+5+1	X, Y, Z, dt, Tw, ISB, n*N

**Table 2:** Combinations coefficients of different fundamental signals in CIF2 and CIF3 for GPS and BDS. As to measurement noise (m), it is defined as $\sigma_{P}=0.3$ m, $\sigma_{L}=0.003$ m in both the Global Positioning System (GPS) and BeiDou Navigation Satellite System (BDS).

Model	System	Signal Combination	$k_{1}$	$k_{2}$	$k_{3}$	ε (Cycle)	Noise (m)
CIF2	GPS	$P_{1}$, $P_{2}$	2.5457	−1.5457	0	297.8	0.8934
		$L_{1}$, $L_{2}$	2.5457	−1.5457	0	2.978	0.0089
	BDS	$P_{1}$, $P_{2}$	2.4872	−1.4872	0	289.8	0.8694
		$L_{1}$, $L_{2}$	2.4872	−1.4872	0	2.898	0.0087
CIF3	GPS	$P_{1}$, $P_{2}$, $P_{5}$	2.3269	−0.3596	−0.9673	254.5	0.7635
		$L_{1}$, $L_{2}$, $L_{5}$	2.3269	−0.3596	−0.9673	2.545	0.0076
	BDS	$P_{1}$, $P_{2}$, $P_{3}$	2.5664	−1.2289	−0.3375	286.5	0.8596
		$L_{1}$, $L_{2}$, $L_{3}$	2.5664	−1.2289	−0.3375	2.865	0.0086

**Table 3:** Error corrections. Differential code bias (DCB).

Error Corrections	Setting
DCB	Multi-GNSS Experiment (MGEX)
Cycle slip	Melbourne-Wubbena detecting (M-W) and Ionosphere Residuals
Clock slip	Guo [33]
Observation weighting	Witchayangkoon [34] and Helmert
Earth rotation	Sagnac effect
Relativistic effects	Xu and Xu [23]
Troposphere	Random walk + Hopfield + Global Mapping Function (GMF)
Antenna phase center	Antenna Phase Center Offsets (PCO)+Antenna Phase Center Variations (PCV)
Phase windup	Corrected
Earth tides correction	Solid/Pole tide

**Table 4:** Parameter settings.

Parameter	Setting
Rinex file	xxxx1000.18o
Precise orbit product	gbm19962.sp3
Precise clock product	gbm19962.clk
Pole shift/ut1-utc	gbm19962.erp
Antenna phase center	igs14.atx
Positioning mode	static
Estimation algorithm	Standard Kalman Filter
Reference coordinate	gbm19962.clk
Sample rate	30 s
Elevation cutoff angle	$5^{\circ }$

**Table 5:** Accuracies for different combined models with the daily solution in North (N), East (E), Up (U) directions (mm).

Model	CEDU			DARW			JFNG			KARR			KZN2		
	N	E	U	N	E	U	N	E	U	N	E	U	N	E	U
UC3-C	16	23	108	14	47	84	27	34	45	15	64	35	24	19	35
CIF3-C	15	19	97	17	50	81	26	34	42	16	70	33	13	24	37
ECIF2-C	17	20	110	5	38	107	30	29	43	6	58	52	14	19	48
CIF2-C	18	29	131	8	36	115	21	22	31	4	55	73	11	25	45
UC3-BDS	33	18	108	18	29	164	13	55	165	23	29	64	29	53	147
CIF3-BDS	33	17	119	21	33	162	11	48	115	23	28	74	26	56	151
CIF2-BDS	33	17	119	19	34	159	11	48	115	23	28	74	25	56	149
CIF2-GPS	18	33	139	8	41	136	20	18	24	4	54	84	11	24	49

**Table 6:** Precisions for different combined models with the daily solution in North, East, Up directions (mm).

Model	CEDU			DARW			JFNG			KARR			KZN2		
	N	E	U	N	E	U	N	E	U	N	E	U	N	E	U
UC3-C	9	10	18	4	12	27	6	9	22	8	22	20	64	13	31
CIF3-C	8	10	20	6	13	33	6	10	18	10	27	18	5	9	29
ECIF2-C	6	8	24	4	16	26	12	15	31	5	17	27	7	16	37
CIF2-C	5	9	36	3	9	23	8	7	20	4	10	33	7	8	34
UC3-BDS	13	17	22	10	17	34	6	12	60	15	11	21	9	20	9
CIF3-BDS	13	17	23	11	18	38	6	7	47	15	10	24	9	19	11
CIF2-BDS	13	17	23	10	18	37	6	7	46	15	10	24	9	20	11
CIF2-GPS	5	10	37	3	9	26	7	6	19	3	9	37	8	7	30

**Table 7:** Mean accuracy, mean precision, mean convergence time, and median convergence time (CT) for different combined models with the daily solution at different stations.

Model	Accuracy (N,E,U) (mm)	Precision (N,E,U) (mm)	Mean CT (s)	Median CT (s)
UC3-C	(19, 38, 61)	(7, 13, 24)	46.6	40
CIF3-C	(18, 39, 58)	(7, 14, 23)	54	55
ECIF2-C	(15, 33, 72)	(7, 14, 29)	58.9	55.5
CIF2-C	(12, 33, 79)	(5, 8, 29)	22.8	13.5
UC3-BDS	(23, 37, 130)	(11, 15, 29)	163.8	179.25
CIF3-BDS	(23, 37, 124)	(10, 14, 28)	190	196
CIF2-BDS	(22, 37, 123)	(22, 37, 123)	191	197.5
CIF2-GPS	(12, 34, 86)	(5, 8, 30)	16.4	15

## Abbreviations

The following abbreviations are used in this manuscript:

BDS	BeiDou Navigation Satellite System
CIF	conventional ionosphere-free model
CIF2	conventional ionosphere-free model with dual-frequency
CIF2-BDS	conventional ionosphere-free model with dual-frequency BDS-only
CIF2-C	conventional ionosphere-free model with dual-frequency BDS+GPS combination
CIF2-GPS	conventional ionosphere-free model with dual-frequency GPS-only
CIF3	conventional ionosphere-free model with triple-frequency
CIF3-BDS	conventional ionosphere-free model with triple-frequency BDS-only
CIF3-C	conventional ionosphere-free model with triple-frequency BDS+GPS combination
CORS	Continuously Operating Reference Stations
CT	convergence time
DCB	differential code bias
DOY	day of year
ECIF2-C	extended CIF2-C model
FCB	fractional cycle bias
GMF	Global Mapping Function
GNSS	Global Navigation Satellite System
GPS	Global Positioning System
ICD	Interface Control Document
IFB	inter-frequency clock bias
ISB	inter-system clock bias
ISC	inter-signal correction
MGEX	Multi-GNSS Experiment
M-W	Melbourne-Wubbena detecting
PCO	Antenna Phase Center Offsets
PCV	Antenna Phase Center Variations
PPP	precise point positioning
TGD	timing group delay
UC	uncombined model
UC2	uncombined model with dual-frequency
UC3	uncombined model with triple-frequency
UC3-BDS	uncombined model based with triple-frequency BDS-only
UC3-C	uncombined model with triple-frequency BDS+GPS combination
UPD	uncalibrated phase delay

## References

[1] Zumberge J., Heflin M., Jefferson D., Watkins M., Webb F.H. (1997). Precise Point Positioning for the efficient and robust analysis of GPS data from large networks. J. Geophys. Res. Solid Earth.

[2] Kouba J., Héroux P. (2001). Precise Point Positioning using IGS orbit and clock products. GPS Solut..

[3] Guo F., Zhang X., Wang J. (2015). Timing group delay and differential code bias corrections for BeiDou positioning. J. Geod..

[4] Hatch R., Jung J., Enge P., Pervan B. (2000). Civilian GPS: The benefits of three frequencies. GPS Solut..

[5] Rho H., Langley R. (2009). The WAAS L5 Signal: An Assessment of Its Behavior and Potential End Use. GPS World.

[6] Montenbruck O., Hugentobler U., Dach R., Steigenberger P., Hauschild A. (2012). Apparent clock variations of the Block IIF-1 (SVN62) GPS satellite. GPS Solut..

[7] Montenbruck O., Hauschild A., Steigenberger P., Hugentobler U., Teunissen P., Nakamura S. (2013). Initial assessment of the COMPASS/BeiDou-2 regional navigation satellite system. GPS Solut..

[8] Zhang X., Wu M., Liu W., Li X., Yu S., Lu C., Wickert J. (2017). Initial assessment of the COMPASS/BeiDou-3: New-generation navigation signals. J. Geod..

[9] Elsobeiey M. (2015). Precise point positioning using triple-frequency GPS measurements. J. Navig..

[10] Guo F., Zhang X., Wang J., Ren X. (2016). Modeling and assessment of triple-frequency BDS precise point positioning. J. Geod..

[11] Li H., Li B., Xiao G., Wang J., Xu T. (2016). Improved method for estimating the inter-frequency satellite clock bias of triple-frequency GPS. GPS Solut..

[12] Pan L., Zhang X., Li X., Liu J., Li X. (2017). Characteristics of inter-frequency clock bias for Block IIF satellites and its effect on triple-frequency GPS precise point positioning. GPS Solut..

[13] Zhao L., Ye S., Song J. (2017). Handling the satellite inter-frequency biases in triple-frequency observations. Adv. Space Res..

[14] Li H., Li B., Lou L., Yang L., Wang J. (2017). Impact of GPS differential code bias in dual-and triple-frequency positioning and satellite clock estimation. GPS Solut..

[15] Pan L., Li X., Zhang X., Li X., Lu C., Zhao Q., Liu J. (2017). Considering inter-frequency clock bias for BDS triple-frequency precise point positioning. Remote. Sens..

[16] Pan L., Zhang X., Li X., Liu J., Guo F., Yuan Y. (2018). GPS inter-frequency clock bias modeling and prediction for real-time precise point positioning. GPS Solut..

[17] Ye S., Zhao L., Song J., Chen D., Jiang W. (2018). Analysis of estimated satellite clock biases and their effects on precise point positioning. GPS Solut..

[18] Guo J., Geng J. (2018). GPS satellite clock determination in case of inter-frequency clock biases for triple-frequency precise point positioning. J. Geod..

[19] El-Mowafy A., Deo M., Rizos C. (2016). On biases in precise point positioning with multi-constellation and multi-frequency GNSS data. Meas. Sci. Technol..

[20] Aggrey J., Bisnath S. Analysis of multi-GNSS PPP initialization using dual-and triple-frequency data. Proceedings of the 2017 International Technical Meeting of The Institute of Navigation.

[21] Teunissen P., Kleusberg A. (1998). GPS for Geodesy.

[22] Leick A., Rapoport L., Tatarnikov D. (2015). GPS Satellite Surveying.

[23] Xu G., Xu Y. (2016). GPS: Theory, Algorithms and Applications.

[24] Montenbruck O., Steigenberger P. (2013). The BeiDou navigation message. J. Glob. Position Syst..

[25] Zhao Q., Guo J., Li M., Qu L., Hu Z., Shi C., Liu J. (2013). Initial results of precise orbit and clock determination for COMPASS navigation satellite system. J. Geod..

[26] Lou Y., Liu Y., Shi C., Yao X., Zheng F. (2014). Precise orbit determination of BeiDou constellation based on BETS and MGEX network. Sci. Rep..

[27] Rao G.S. (2007). GPS satellite and receiver instrumental biases estimation using least squares method for accurate ionosphere modelling. J. Earth Syst. Sci..

[28] Li M., Qu L., Zhao Q., Guo J., Su X., Li X. (2014). Precise point positioning with the BeiDou navigation satellite system. Sensors.

[29] Montenbruck O., Hauschild A., Steigenberger P. (2014). Differential Code Bias Estimation using Multi-GNSS Observations and Global Ionosphere Maps. Navig. J. Inst. Navig..

[30] Schaer S., Steigenberger P. Determination and use of GPS differential code bias values. Proceedings of the IGS Workshop.

[31] Schaer S. (2012). Overview of GNSS Biases.

[32] Zhao Q., Wang G., Liu Z., Hu Z., Dai Z., Liu J. (2016). Analysis of BeiDou satellite measurements with code multipath and geometry-free ionosphere-free combinations. Sensors.

[33] Guo F. (2016). Theory and Methodology of Quality Control and Quality Analysis for GPS Precise Point Positioning.

[34] Witchayangkoon B. (2000). Elements of GPS Precise Point Positioning. Ph.D. Thesis.

[35] Shen X. (2002). Improving Ambiguity Convergence in Carrier Phase-based Precise Point Positioning. Ph.D. Thesis.

[36] Liu P., Qin H., Cong L. (2019). The Feasible Combining Observation Models and Equivalence in Dual-Frequency Precise Point Positioning. IEEE Access.

[37] Rizos C., Montenbruck O., Weber R., Weber G., Hugentobler U. The IGS MGEX experiment as a milestone for a comprehensive multi-GNSS service. Proceedings of the ION 2013 Pacific PNT Meeting.

[38] Choy S., Bisnath S., Rizos C. (2017). Uncovering common misconceptions in GNSS Precise Point Positioning and its future prospect. GPS Solut..

